# Aberrant effective connectivity is associated with positive symptoms in first-episode schizophrenia

**DOI:** 10.1016/j.nicl.2020.102444

**Published:** 2020-09-22

**Authors:** Martin J. Dietz, Yuan Zhou, Lotte Veddum, Christopher D. Frith, Vibeke F. Bliksted

**Affiliations:** a Center of Functionally Integrative Neuroscience, Institute of Clinical Medicine, Aarhus University, Denmark; b CAS Key Laboratory of Behavioral Science, Institute of Psychology, Beijing 100101, PR China; c Department of Psychology, University of Chinese Academy of Sciences, Beijing 100049, PR China; d Psychosis Research Unit, Aarhus University Hospital, Denmark; e Institute of Clinical Medicine, Aarhus University, Denmark; f The Wellcome Centre for Human Neuroimaging, University College London, United Kingdom; g Interacting Minds Centre, Aarhus University, Denmark

**Keywords:** Schizophrenia, Social cognition, Brain-symptom mapping, Dynamic causal modelling (DCM), Parametric empirical Bayes (PEB)

## Abstract

Schizophrenia is a neurodevelopmental psychiatric disorder thought to result from synaptic dysfunction that affects distributed brain connectivity, rather than any particular brain region. While symptomatology is traditionally divided into positive and negative symptoms, abnormal social cognition is now recognized a key component of schizophrenia. Nonetheless, we are still lacking a mechanistic understanding of effective brain connectivity in schizophrenia during social cognition and how it relates to clinical symptomatology. To address this question, we used fMRI and dynamic causal modelling (DCM) to test for abnormal brain connectivity in twenty-four patients with first-episode schizophrenia (FES) compared to twenty-five matched controls performing the Human Connectome Project (HCP) social cognition paradigm. Patients had not received regular therapeutic antipsychotics, but were not completely drug naïve. Whilst patients were less accurate than controls in judging social stimuli from non-social stimuli, our results revealed an increase in feedforward connectivity from motion-sensitive V5 to posterior superior temporal sulcus (pSTS) in patients compared to matched controls. At the same time, patients with a higher degree of positive symptoms had more disinhibition within pSTS, a region computationally involved in social cognition. We interpret these findings the framework of active inference, where increased feedforward connectivity may encode aberrant prediction errors from V5 to pSTS and local disinhibition within pSTS may reflect aberrant encoding of the precision of cortical representations about social stimuli.

## Highlights

• We use DCM in patients newly diagnosed with schizophrenia.
• Patients were naïve to therapeutic antipsychotics, but not completely drug naïve.
• Patients have stronger feedforward connectivity than matched healthy controls.
• Stronger positive symptoms are associated with disinhibition in the temporal lobe.
• In active inference, this may relate to aberrant precision and prediction errors.

## 1 Introduction

Schizophrenia is a neurodevelopmental psychiatric disorder that affects about 1% of the population worldwide (Javitt, 2010). The symptoms of first-episode schizophrenia manifest across an age range from adolescence to early adulthood (Jongsma et al., 2019). The highest incidence of first-episode schizophrenia is around age 22 (Bergen et al., 2014; Pedersen et al., 2014), whereas early-onset schizophrenia (EOS) (age 14–18) is very rare, has a more severe prognosis and many EOS patients are rediagnosed later in life (Clemmensen et al., 2012, Driver et al., 2013). While symptomatology is traditionally divided into positive and negative symptoms on the one hand, and cognitive deficits on the other, abnormal social cognition is recognized a key component of schizophrenia (Friston and Frith, 2015). Recent research has shown that patients with high levels of both positive and negative symptoms, or negative symptoms alone, have more profound deficits in theory of mind compared to patients with only positive symptoms (Bliksted et al., 2016, Kästner et al., 2015). While negative symptoms have been associated with hypo-mentalizing similar to autism (Bliksted et al., 2017), positive symptoms have been associated with hyper-mentalizing, in particular delusions and paranoia (Ciaramidaro et al., 2015). Importantly, it has been suggested that patients with schizophrenia could be switching between hyper- and hypo-mentalizing, depending on social context (Bliksted et al., 2016, Bliksted et al., 2019).

Theory of mind refers to the belief that other people have minds different from our own (Frith, 1992). From a computational perspective, it is an agent’s ability to generate an internal model of another agent’s beliefs about the world. This internal model is necessary to explain other agents’ behavior in terms of their intentions, goals and desires (Friston and Frith, 2015). Without a generative model of mental states to explain observed behavior, one wouldn’t know that a smile (behavior) is caused by happiness (mental state) or that tears can be happy or sad, depending on which theory of mind (model) we adopt. One of the most validated and widely used theory-of-mind tasks is the “Animated Triangles” task (Abell et al., 2000, Blakemore, 2008, Pinkham and Harvey, 2013). Brain mapping studies using fMRI have consistently associated theory of mind with increased activation of the posterior superior temporal sulcus (pSTS) and the medial prefrontal cortex (Blakemore, 2008, Green et al., 2015). This paradigm has also been used to identify abnormal BOLD activation in patients with schizophrenia compared to healthy controls (Bliksted et al., 2019, Das et al., 2012a, Das et al., 2012b, Koelkebeck et al., 2013; A. Pedersen et al., 2012). However, in contrast to healthy subjects with normal neurodevelopment, brain activation in patients with schizophrenia has been inconsistent. While Das *et al.* found reduced activation of the right superior temporal gyrus (STG), the temporo-parietal junction (TPJ) and bilateral inferior frontal gyri (IFG) in male patients with established schizophrenia (Das et al., 2012b, Das et al., 2012a), Martin *et al.* showed increased activation of bilateral IFG, left STG and left caudate nucleus (Martin et al., 2016) in chronic, medicated patients. In contrast, Bliksted *et al.* found that largely drug-naïve first-episode schizophrenia (FES) patients hyper-mentalized during non-social stimuli, accompanied by increased activation of the anterior medial prefrontal cortex (Bliksted et al., 2019).

Schizophrenia is a neurodevelopmental disorder thought to emerge with synaptic dysfunction (Adams et al., 2013, McCutcheon et al., 2019, Stephan et al., 2009). A variety of studies using non-invasive electrophysiology (EEG and MEG) have identified abnormal post-synaptic responses to both auditory stimuli (Avissar et al., 2018, Ranlund et al., 2015, Umbricht and Krljes, 2005), visual stimuli (Grent-'t-Jong et al., 2016, Sun et al., 2013, Tan et al., 2013, Tsuchimoto et al., 2011) and tactile stimuli (Huang et al., 2009, Reite et al., 2003) in patients with schizophrenia compared to healthy controls. In addition to the abnormal neurophysiology that inherits from synaptic dysfunction, there is some evidence of widespread white-matter abnormalities that could impair axonal conduction between brain areas (Kelly et al., 2018, Shepherd et al., 2012, Yao et al., 2013). However, we are still lacking a mechanistic understanding of effective brain connectivity in patients with first-episode schizophrenia.

To this end, we used functional magnetic resonance imaging (fMRI) and dynamic causal modelling (DCM) to test for abnormal brain connectivity in twenty-four patients with first-episode schizophrenia (FES) compared to twenty-five matched controls during the HCP social cognition paradigm (Barch et al., 2013). First, we use Bayesian model reduction and parametric empirical Bayes (PEB) (Friston et al., 2015) to test for aberrant feedforward, feedback and intrinsic (inhibitory) connectivity in FES patients. Finally, we use PEB and Bayesian model comparison to test how these connectivity estimates are differentially related to positive and negative symptomatology.

## 2 Material and methods

### 2.1 Patients

We initially recruited 31 patients from the OPUS Clinic, a first-episode schizophrenia clinic at Aarhus University Hospital, Aarhus, Denmark. While each patient met the ICD-10 criteria for schizophrenia, they had no history of neurological disorder or severe head trauma according to ICD-10, nor did they have an ICD-10 diagnosis of drug- or alcohol dependency. Patients were excluded if they had an estimated premorbid IQ < 70 based on their history or if they were not able to understand spoken Danish sufficiently well to comprehend the testing procedures. Seven patients were excluded from the fMRI experiment due to dental braces (N = 3), pregnancy (N = 1) and several no-shows (N = 3). Finally, 24 first-episode patients were included in the fMRI experiment. Given that our patients were newly diagnosed, most of them did not receive regular doses of antipsychotic medication at a therapeutic level that could be converted to standard chlorpromazine equivalents (Woods, 2005). Some of the patients received their initial depot injection a few days before the MRI scan, so that a stable, therapeutic concentration was not yet expected. Other patients only agreed to take very low doses of antipsychotics used as an *ad hoc* sedative in order to fall asleep (e.g. 25 mg Quetiapine, where 750 mg is the expected clinical antipsychotic dose). See Table 1 for a summary of the patients' medication history.

**Table 1:** Medication history of first-episode schizophrenia patients.

N	Atypical antipsychotics			Antidepressants	Other medication
	Depot	Standard	Low		
5	*				
4				*	
2			*		
2	*		*		
2	*				*
2					*
1		*	*	*	
1		*		*	
1			*		*
1	*	*			
1	*			*	*
1		*		*	*
1		*			

Depot: monthly injected therapeutic dose, recently initiated

Standard: oral therapeutic dose

Low**:** dose below antipsychotic effect, used as *ad hoc* sedative

Other**:** sleeping pills, analgesics, antihypotensives, anxiolytics, mood stabilizers

**Table 2:** Demographics, psychopathology, IQ, and social cognition.

	Schizophrenia (N = 24)	Healthy controls (N = 25)	Statistics	*P*
Age, mean (95% CI)	25.21 (23.35, 27.07)	24.6 (22.78, 26.42)	z = -0.62	0.53^1^
Females, N (%)	7(39)	11(61)	Chi^2^(1) = 1.16	0.28^2^
Years of education,mean (95% CI)	15.9 (14.91, 16.89)	14.60 (13.59, 15.62)	z = 1.61	0.11^1^
Current occupation, N (%)			Chi^2^(5) = 18.06	0.003^2^(0.001)^3^
Unemployed	6(25)	1(4)		
Work	7(29)	5(20)		
Student	5(21)	19(76)		
Sick leave	3(13)	0(0)		
Pension	1(4)	0(0)		
Other	2(8)	0(0)		
SANS, mean (95% CI)	8.17 (7.02, 9.31)	1.32 (0.20, 2.44)	z = -5.55	<0.0001^1^
SAPS, mean (95% CI)	7.08 (5.97, 8.20)	0.28 (-0.81, 1.37)	z = -5–54	<0.0001^1^
PSP	55.03 (48.93, 61.23)	86.32 (83.20, 89.44)	t(34) = 9.37	<0.001^4^
GAF-F	56.39 (51.78, 61.00)	86.56 (82.14, 90.98)	z = 5.56	<0.001^1^
WAIS-III (estimated IQ)	92.96 (84.49, 101.43)	97.4 (86.19, 108.61)	t(47) = 0.65	0.52^4^
ATT				
Intentionality – ToM	14 (12.67 15.33)	15.24 (13.94, 16.54)	z = 1.31	0.19^1^
Intentionality - Random	0.75 (0.28, 1.22)	1.67 (0.64, 2.70)	z = -1.58	0.11^1^
Accuracy - ToM	8.04 (7.26, 8.82)	8.88 (8.12, 9.64)	z = 1.47	0.14^1^
Acuracy - Random	0.75 (0.26, 1.24)	2.27 (0.83, 3.71)	z = -1.96	0.05^1^

SANS: Scale for Assessment of Negative Symptoms

SAPS: Scale for Assessment of Positive Symptoms

PSP: Personal and Social Performance scale

GAF-F: Global Assessment of Functioning – level of social functioning

WAIS-III: Wechsler Adult Intelligence Scale-III (Block Design, Vocabulary)

ATT: Animated Triangles Task

1 Mann-Whitney *U* test, ^2^ Chi-squared test, ^3^ Fisher's exact test, ^4^ Student's *t*-test

### 2.2 Healthy controls

We initially recruited 29 healthy controls. Exclusion criteria were the same as for the patients, except that controls were excluded if they or a first-degree relative had an ICD-10 diagnosis, or if a diagnosis was confirmed during the Present State Examination (PSE) interview (ICD-10, WHO). Four controls were excluded from the fMRI experiment on the day of scanning: two had dental braces and two left the study prematurely. FES patients and healthy controls were intended matched on age, gender, educational level (last commenced education), and parental socioeconomic status (SES). However, we did not succeed completely with this strategy, so the two groups ended up being matched on a group level. Finally, 25 healthy controls were included in the fMRI study.

### 2.3 Psychopathology and social functioning

First-episode schizophrenia patients were interviewed by a psychiatrist with the Present State Examination interview regarding schizophrenia and drug dependency (WHO, 1994). All FES patients and healthy controls were rated with the Scale for the Assessment of Negative symptoms (SANS) and the Scale for the Assessment of Positive Symptoms (SAPS) (Andreasen, 1984a, Andreasen, 1984b). Level of psychosocial functioning was measured using the Global Assessment of Functioning (GAF-F) (Startup et al., 2002) and the Personal and Social Performance Scale (PSP) (Nasrallah et al., 2008).

### 2.4 Intelligence and social cognition

We estimated intelligence using two subtests from the Wechsler Adult Intelligence Scale (WAIS-III) (Wechsler, 1997). The two subtests were chosen based on their high correlation with the total WAIS-III IQ-score: Block Design and Vocabulary. Theory of mind ability was evaluated diagnostically using the “Animated Triangles” task (Abell et al., 2000). In the “random” condition, the triangles move randomly about. In the “theory of mind” condition, the animated triangles move in a coordinated fashion that resembles a social interaction, a scenario that normally developing individuals consistently explain using theory of mind (Abell et al., 2000). There are four clips of each type of animation with 38–41 s duration. After each animation, the participants were asked to describe what they thought was happening and their answers were scored regarding degree of mental state attribution (range 0–5) and appropriateness of their description (range 0–3) as outlined in Appendix 2 of (Castelli et al., 2000). Each answer was scored by two clinical psychologists (LV and VB) and the ordinal scores were summed within category for statistical analysis. Using the Mann-Whitney *U* test, we then tested for differences in the distribution of scores between patients with schizophrenia and healthy controls. Intraclass correlation (ICC) in a two-way random-effects model showed absolute agreement of intentionality scores in the “random” condition (ICC = 0.97, 95% CI (0.93; 0.99), *P* < 0.0001) and the “theory of mind” condition (ICC = 0.96 95% CI (0.89; 0.98), *P* < 0.001) and absolute agreement of appropriateness scores in the “random” condition (ICC = 0.97, 95% CI (0.92; 0.99), *P* < 0.001) and the “theory of mind” condition (ICC = 0.96, 95% CI (0.91; 0.98), *P* < 0.001) among the two ratings.

### 2.5 fMRI paradigm

**Fig. 1:**
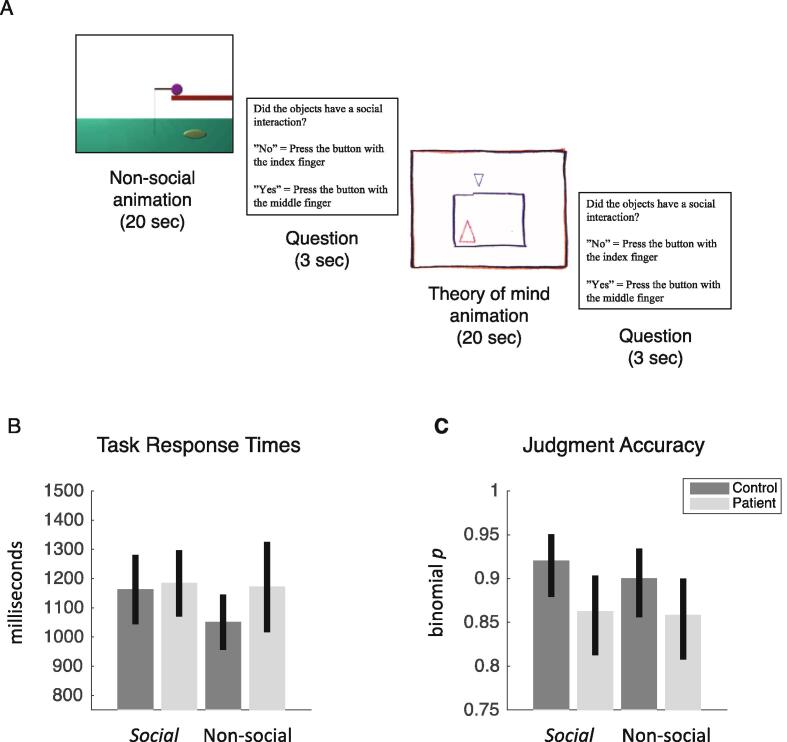
Judgment of social versus non-social stimuli (A) Social cognition paradigm (B) Response times with 95% confidence intervals (C) Judgment accuracy with 95% confidence intervals: patients with schizophrenia were less accurate in detecting social scenarios than healthy controls, with no consistent difference between groups when judging non-social scenarios.

To test for abnormal brain connectivity during social cognition, we used the social cognition paradigm from the Human Connectome Project (HCP) (Barch et al., 2013) with permission from the WU-Minn HCP consortium (http://www.humanconnectome.org/). Visual stimuli consist of animated sequences of 20 s duration showing geometric shapes (triangles, squares and circles) that move about in either a coordinated fashion that resembles a social interaction among individuals (social motion) or in a random fashion (non-social motion). Participants were presented with 10 sequences of social scenarios and 10 sequences of non-social scenarios in randomized order. After each stimulus sequence, participants were asked whether they had perceived a social interaction, answering ‘yes’ with their right index finger or ‘no’ with their right middle finger. The response period lasted 3 s. The paradigm was presented with E-prime 2.0 (Psychology Software Tools, Inc.) and projected onto a screen at the back of the MRI bore that participants viewed through a mirror mounted on top of the radio frequency coil. Each block of stimulus and response was followed by 15 s of visual fixation (see Fig. 1).

### 2.6 Ethics statement

All participants received written and verbal information about the project and a written informed consent was obtained before inclusion. The study was approved by the Central Denmark Region Committee on Health Research Ethics (Ref: 1–16-02–87-15) and the Danish Data Protection Agency. The project complied with the Helsinki-II-declaration.

### 2.7 fMRI acquisition

T2*-weighted echo planar images (EPI) were acquired on a 3 T Siemens Magnetom Trio using a 32-channel RF head coil at the Center of Functionally Integrative Neuroscience, Aarhus University, Denmark. Each volume consisted of 40 slices with 3 mm thickness acquired in descending order with repetition time (TR) = 2 sec, echo time (TE) = 27 ms, flip angle = 90°, field of view (FOV) = 192 × 192 mm and in-plane resolution = 64 × 64. The subjects’ head was fixated with soft cushions to minimize head movement during the experiment.

### 2.8 fMRI analysis

fMRI data were analyzed using Statistical Parametric Mapping (SPM12, revision 6906). Echo-planar (EPI) images were resampled to 2 mm^3^ voxels, realigned within subject and spatially normalized to MNI space using the ICBM template of European brains. The time-series were high-pass filtered at 1/128 s using a discrete cosine set and temporal correlations were modelled using a first-order autoregressive (AR(1)) model. Social and non-social motion conditions were modelled as boxcar regressors convolved with a canonical HRF and fitted to the BOLD time-series using a general linear model (Worsley and Friston, 1995). Visual fixation periods were not modelled and hence constituted an implicit baseline. Finally, the scan-to-scan realignment parameters (translations and rotations) were included in the GLM to adjust for the effects of head movement. Head movement did not differ between patients and healthy controls (RMS displacement: *F*(1, 47) = 0.54, *p* = 0.47). We created contrast images for each patient and control testing for visual motion in general (both social and non-social versus fixation) and the difference in activation between social and non-social stimuli. Finally, contrast images were smoothed with a 6-mm FWHM Gaussian kernel and used as summary statistics in a random-effects analysis using one-sample *t*-tests within patients and controls, separately. To identify regions active both during the perception of visual motion in general and during social motion in particular, we used a conjunction analysis to test for a conjunction of *t*-tests (global null). This corresponds to masking one significant contrast with another to identify an overlap of significant activations (Friston et al., 2005). We thus tested for visual motion in general (conjunction) and for social compared to non-social stimuli to identify effects in each group separately, differences between groups and, finally, commonalities across groups. All statistical tests were thresholded at *p* < 0.05, family-wise error (FWE) whole-brain corrected for multiple comparisons using random field theory (Worsley et al., 1996).

### 2.9 Dynamic causal modelling of effective connectivity

We used a two-state dynamic causal model (DCM) for fMRI (DCM12, revision 6755) to estimate the effective connectivity within and between brain areas, given observed haemodynamic measurements (Friston et al., 2003). While one-state DCM for fMRI is used to model extrinsic connections only, two-state DCM models both extrinsic connections between regions as excitatory forward and backward connections and intrinsic connectivity within each region in terms of one inhibitory population and one excitatory population of neurons. This allows us to model the intrinsic connectivity within each cortical area as an increase or decrease in cortical inhibition (Marreiros et al., 2008). We summarised the BOLD signal in each participant using the first eigenvariate (principal component) of voxels within a sphere of 8 mm radius centred on each participant’s local maximum. This subject-specific local maximum was identified within a sphere of 20 mm radius centred on the peak of the group effect. The network derived empirically from the group-level fMRI result comprised motion-sensitive area V5 and posterior superior temporal sulcus p(STS) in the right hemisphere (see Table 3.). The hemodynamic responses to all visual motion (social and non-social stimuli) were mean-centred and modelled as a driving input to area V5 (C-matrix). Using parametric modulation of the regressor encoding all visual motion, the responses to social compared to non-social stimuli were modelled as a modulation (increase or decrease) of the intrinsic and extrinsic connection strengths (B-matrix) in relation to the average connectivity estimated from the mean-centred responses to all visual motion (A-matrix).

**Table 3:** Brain mapping commonalities among patients and controls: difference between social and non-social stimuli in regions that were also activated by visual motion in general (conjunction analysis).

*T s*tatistic	MNI coordinate	Anatomical region	Probabilistic atlas^1^
16.12	[46–68 0]	Right middle temporal Gyrus	hOc5 (V5) 52% hOc4la 47%
5.61	[54–54 14]	Right superior temporal sulcus	PGa (IPL) 45% PGp (IPL) 17%

1 Anatomical classification using the SPM anatomy toolbox (Eickhoff et al., 2005)

We analysed the intrinsic and extrinsic connectivity between V5 and pSTS under four alternative hypotheses (see Fig. 3 for a schematic). The first hypothesis was formulated as a full DCM where (1) both extrinsic connections between V5 and pSTS and intrinsic connections within V5 and pSTS encode the differences between experimental conditions. The second hypothesis was formulated as a reduced model where (2) only extrinsic connections between V5 and pSTS encode the differences between experimental conditions. The third hypothesis was formulated as a reduced model where (3) only the feedforward connection from V5 and pSTS encodes the differences between experimental conditions. Finally, a null model encoded the belief that (4) no connections change between conditions.

### 2.10 Bayesian model inversion

We then inverted the full dynamic causal model encoding the first hypothesis for each patient and control using variational Laplace (Friston et al., 2007). This provides both the posterior distribution of the connection strengths and the free-energy approximation to the marginal likelihood of the model itself, known as the model evidence. The free parameters and the Bayesian model evidence of each reduced model were then estimated using Bayesian model reduction under the Laplace assumption (Friston et al., 2015).

In variational Laplace, the conditional expectations and covariance of the (multivariate) approximate posterior distribution q(ϑ|y,m) are estimated iteratively by maximizing a lower bound on the logarithm of the model evidence lnp(y|m). This optimization uses Fisher scoring to maximize the (negative) variational free energy F of the model

$$F=\underset{\text{accuracy}}{\underbrace{{\text{E}}_{q}\left[\ln p\left(y|\vartheta ,m\right)\right]}}-\underset{\text{complexity}}{\underbrace{{\text{D}}_{\mathrm{KL}}\left[q\left(\vartheta |y,m\right)\;||\;p\left(\vartheta |m\right)\right]}}$$

where ${\text{E}}_{q}\left[\cdot \right]$ is the expectation under the approximate posterior density q(ϑ|y,m) and ${\text{D}}_{\mathrm{KL}}\left[\cdot \right]$ is the Kullback-Leibner divergence between the approximate posterior and prior probability densities. To understand why the free energy is useful for model comparison, we can decompose it into accuracy and complexity terms. The accuracy is the expected log-likelihood of the data, given the model parameters, and scores the goodness-of-fit of a model. The complexity penalises models that overfit the data by favouring models with low posterior correlation among the parameters. In other words, the free energy penalizes models with high parameter redundancy. The formulation of the complexity as a KL divergence rests on the assumption that the posterior density should not have to move too far from the prior to accommodate the data.

### 2.11 Parametric empirical Bayesian (PEB) analysis of group effects

Having estimated the connection strengths and model evidence at the first level, we then used parametric empirical Bayes (PEB) to identify increases or decreases in connection strengths at the group level. PEB is a hierarchical Bayesian model in which empirical priors on the connection strengths at the single-subject level are estimated empirically using a Bayesian general linear model at the group level (Friston et al., 2015). Unlike classical inference such as ANOVA, this hierarchical Bayesian model allows us to identify commonalities and differences in connection strengths at the group level, taking into account not only the mean of parameter estimates at the single-subject level, but also their variance. This means that subjects with more precise parameter estimates have greater influence on group-level parameters, whereas subjects whose parameters are surrounded by more uncertainty are down-weighted (Zeidman et al., 2019). The advantage of parametric empirical Bayes is that it provides both the posterior distribution of the connection strengths at the group level and the marginal likelihood or Bayesian model evidence of the PEB model itself for Bayesian model comparison of alternative hypotheses. We then compared our alternative hypotheses using both random-effects Bayesian model selection (Penny, 2012, Rigoux et al., 2014) of dynamic causal models at the single-subject level and fixed-effects Bayesian model comparison of PEB-DCMs at the group level.

Finally, we used Bayesian model comparison of PEB models to adjudicate between two alternative hypotheses about the relation between effective connectivity and clinical symptomatology using Bayesian linear regression. In this way, we are able to disambiguate between positive and negative symptoms as the best explanation of patient variability in synaptic efficacy. Normally, one would include medication dose for antipsychotics as a nuisance regressor in the regression model. However, given that all patients were newly diagnosed and did not yet receive standard antipsychotic treatment at a therapeutic level, conversion of their heterogeneous medication to standard chlorpromazine equivalents was not feasible (Woods, 2005). Hence, we were not able to reliably adjust the regression models of positive and negative symptoms for standard doses of antipsychotics.

### 2.12 Software note

The scripts used to reproduce the original results are available from https://github.com/martinjdietz/Publications/tree/master/NICL-2020.

## 3 Results

### 3.1 Demographics, psychopathology, intelligence, and social cognition

Given that patients and controls were matched with regard to age, gender, educational level (last commenced education), and parental socioeconomic status (SES), we did not observe differences between groups in estimated IQ and, to our surprise, no differences in social cognition (Table 2).

### 3.2 Behavioural results of fMRI paradigm

We first analysed response times within healthy controls and FES patients separately. Healthy controls were slower when judging social compared to non-social stimuli (*t*(24) = 2.26, *p* = 0.01, Cohen’s *d* = 0.41, two-tailed *t*-test). In contrast, FES patients showed no difference in response times when judging between social and non-social stimuli (*t*(23) = -0.35, *p* = 0.7, two-tailed *t*-test). We then compared response times between patients and healthy controls. There was no evidence of a difference between groups for social stimuli (*t*(47) = -0.25, *p* = 0.8, two-tailed *t*-test), nor for non-social stimuli (*t*(47) = -1.6, *p* = 0.1, two-tailed *t*-test).

We then tested for a difference in task accuracy using McNemar’s *Chi^2^*-test. This tests for a difference in proportions of accurately judged scenarios within each group separately. Neither healthy controls (*Chi^2^*(1) = 1.08, *p* = 0.3), nor patients with schizophrenia (*Chi^2^*(1) = 0.03, *p* = 0.9) showed evidence of a difference in judgment accuracy between social and non-social stimuli. We then tested for a difference in judgment accuracy between patients and controls using Pearson’s *Chi^2^*-test. This revealed that healthy controls were more accurate in judging social motion than patients with schizophrenia (*Chi^2^*(1) = 4.2, *p* = 0.04, Cramer’s *phi* = 0.09). In contrast, there was no evidence of a difference in accuracy between patients and controls when judging non-social motion (*Chi^2^*(1) = 2, *p* = 0.2).

### 3.3 fMRI brain mapping in healthy controls

**Fig. 2:**
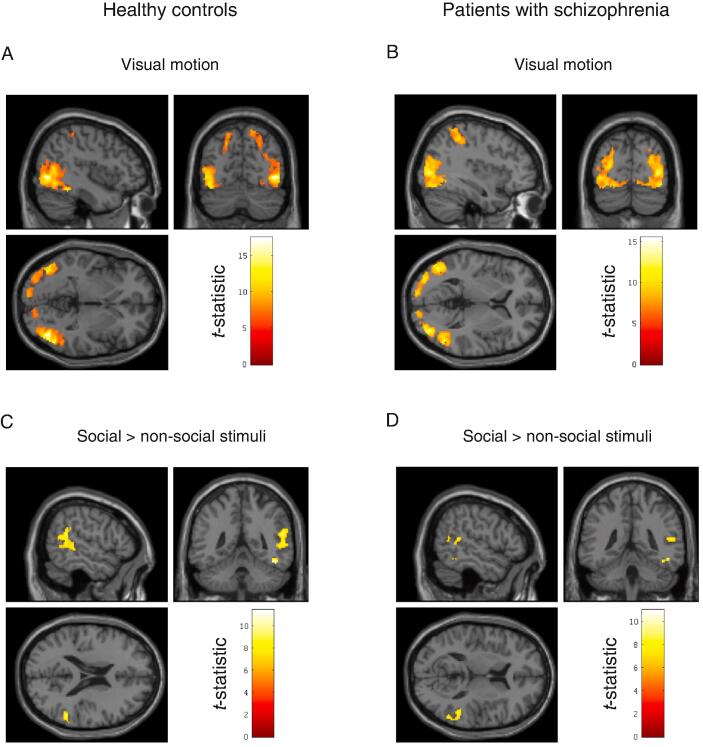
fMRI brain mapping in healthy controls and FES patients (A) Visual motion in healthy controls (B) Visual motion in patients with schizophrenia (C) Social > non-social stimuli in healthy controls (D) Social > non-social stimuli in patients with schizophrenia. Statistical *t*-maps are thresholded at *p* < 0.05, FWE-corrected for multiple comparisons and rendered on a single-subject structural MRI in MNI space. See main text for MNI coordinates.

When healthy controls perceived motion in general (social and non-social) they had increased activation in motion-sensitive area V5 in the right hemisphere with peak at MNI coordinate [44–70 −2], *T*(24) = 17.41, *P_FWE_* < 0.0001, as well as V5 in the left hemisphere with peak at MNI coordinate [-44–72 −10], *T*(24) = 13.42, *P_FWE_* < 0.0001. We also observed increased activation in the left superior parietal lobule (SPL) with peak at MNI coordinate [-30–50 56], *T*(24) = 7.69, *P_FWE_* < 0.0001. In contrast, when healthy controls perceived social motion compared to non-social motion, they had increased activation in posterior inferior temporal gyrus with peak at MNI coordinate [48–50 −18], *T*(24) = 11.40, *P_FWE_* < 0.0001 and posterior superior temporal sulcus (pSTS) with peak at MNI coordinate [56–46 20], *T*(24) = 9.40, *P_FWE_* < 0.0001 (Fig. 2).

**Fig. 3:**
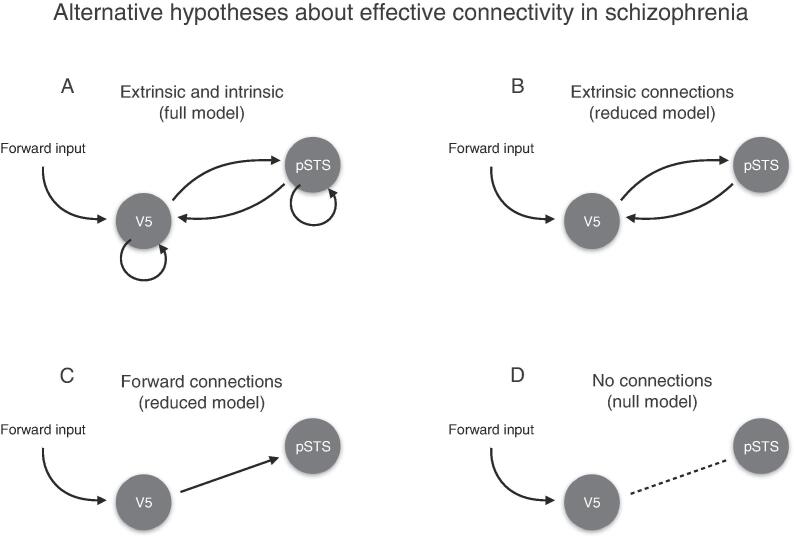
Alternative hypotheses about effective connectivity in patients with first-episode schizophrenia (A) Full model with free parameters on extrinsic and intrinsic connections (B) Reduced model with free parameters on extrinsic connections (C) Reduced model with a free parameter the on feedforwards connection only (D) Null model with no connections between V5 and pSTS.

### 3.4 fMRI brain mapping in patients with first-episode schizophrenia

When patients with first-episode schizophrenia perceived motion in general (social and non-social) they had increased activation in motion-sensitive area V5 in the right hemisphere with peak at MNI coordinate [38–80 4], *T*(23) = 13.40, *P_FWE_* < 0.0001, as well as V5 in the left hemisphere with peak at MNI coordinate [-48–72 0], *T*(23) = 15.45, *P_FWE_* < 0.0001. We also observed increased activation in the right superior parietal lobule (SPL) with peak at MNI coordinate [10–58 60], *T*(23) = 7.24, *P_FWE_* < 0.0001. In contrast, when patients perceived social compared to non-social stimuli, they had increased activation in posterior inferior temporal gyrus with peak at MNI coordinate [46–52 −14], *T*(23) = 9.30, *P_FWE_* < 0.0001 and posterior superior temporal sulcus (pSTS) with peak at MNI coordinate [54–42 12], *T*(23) = 8.05, *P_FWE_* < 0.002. We also observed increased activation in area V4 in right inferior occipital gyrus with peak at MNI coordinate [30–92 −2], *T*(23) = 10.96, *P_FWE_* < 0.0001. There were no differences in BOLD amplitude between patients and controls at a standard family-wise error rate of *P_FWE_* < 0.05 (Fig. 2).

### 3.5 Brain mapping commonalities among patients and controls

We then used a conjunction analysis to identify regions active both during the perception of visual motion in general (social and non-social) as well as during social motion in particular (social minus non-social) in both patients and controls. This revealed two main clusters in the right hemisphere centred on motion-sensitive area V5 and the posterior superior temporal sulcus (pSTS) summarized in Table 3. Importantly, the activation of the pSTS during social motion conforms to the anatomical findings in the literature (Schurz et al., 2017). The time-series in these two regions, common to both patients and controls, were then used for dynamic causal modelling.

### 3.6 Effective connectivity between V5 and pSTS in healthy controls

**Fig. 4:**
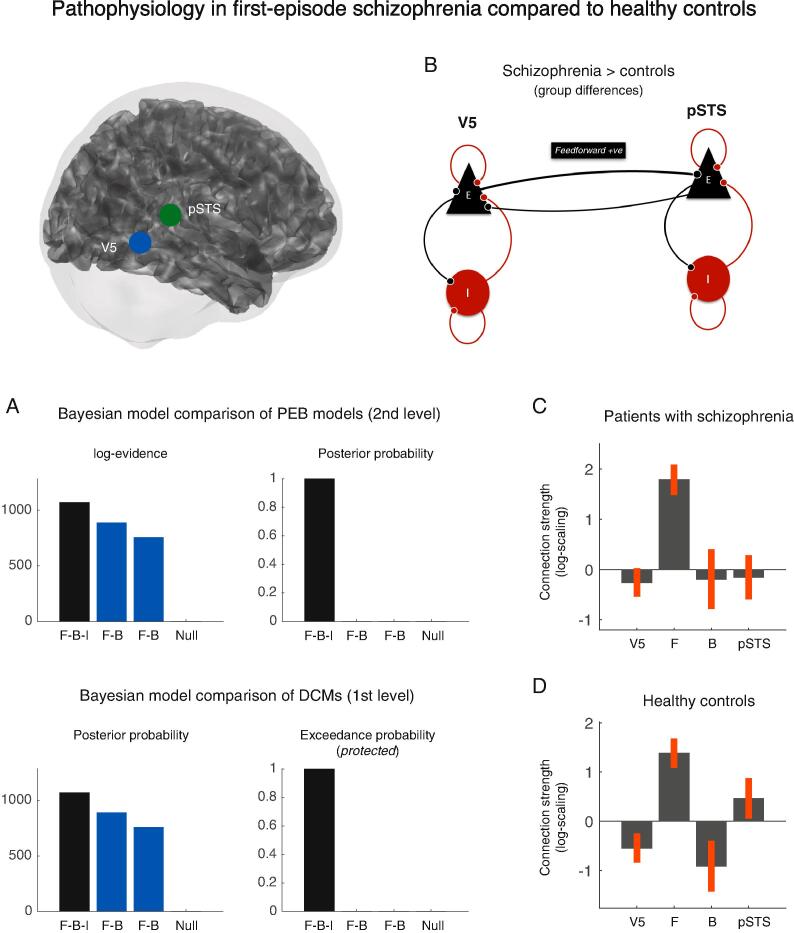
Pathophysiology in first-episode schizophrenia compared to healthy controls (A) Bayesian model comparison of PEB models (2nd-level models) and random-effects Bayesian model comparison of DCMs (1st-level models) (B) Dynamic causal model showing stronger feedforward connectivity in patients compared to healthy controls (C) Connection strengths in patients with first-episode schizophrenia during social > non-social stimuli and (D) connection strengths in healthy controls.

Using dynamic causal modelling (DCM) and parametric empirical Bayes (PEB), we analysed the strength of extrinsic connectivity between V5 and pSTS in the right hemisphere, as well as inhibitory connectivity within each area. While the responses to motion in general were modelled as a driving input to V5, the responses to social versus non-social stimuli were modelled as a modulation (increase or decrease) of the intrinsic and extrinsic connection strengths and hence constituted the experimental effects-of-interest. Random-effects Bayesian model comparison of DCMs (Penny et al., 2010) revealed that the cortical network with changes in both extrinsic and intrinsic connections had the highest posterior probability in healthy controls (Posterior model probability > 0.85 and protected exceedance probability > 0.99). This was confirmed by a Bayesian model comparison of the PEB models at the group level (Posterior model probability > 0.99). Within this network, healthy controls had an increase in feedforward connectivity from V5 to pSTS (Posterior probability > 0.99) and a decrease in feedback connectivity (Posterior probability > 0.99). At the same time, there was a decrease in intrinsic (inhibitory) coupling within V5 (Posterior probability > 0.99) and a concomitant increase in intrinsic (inhibitory) coupling within pSTS (Posterior probability > 0.99) (Fig. 4).

### 3.7 Aberrant effective connectivity in patients with schizophrenia compared to healthy controls

Using parametric empirical Bayes (PEB), we then tested for differences in the strength of extrinsic and intrinsic connectivity between patients with schizophrenia and healthy controls. Again, random-effects Bayesian model comparison of DCMs (Penny et al., 2010) revealed that the full model had the highest posterior probability across patients and controls (Posterior probability > 0.89 and protected exceedance probability > 0.999). This was confirmed by a Bayesian model comparison of PEB models at the group level (Posterior probability > 0.99). Within this network, patients had increased feedforward connectivity when they perceived social stimuli compared to the healthy controls (Posterior probability > 0.97) (Fig. 4).

### 3.8 Patients with stronger positive symptoms have more disinhibition within pSTS

**Fig. 5:**
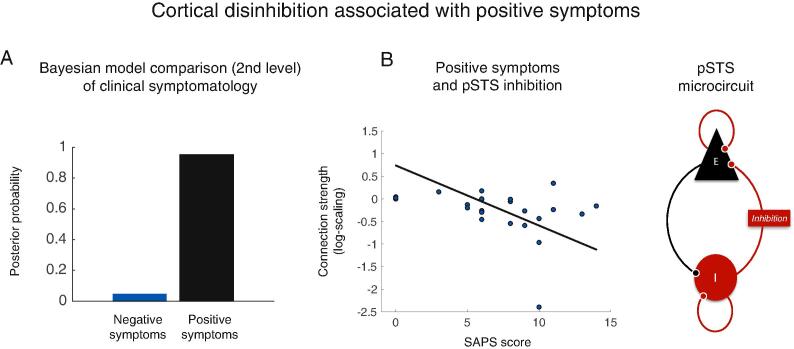
Intrinsic connectivity associated with positive symptoms in first-episode schizophrenia (A) Bayesian model comparison of PEB models of clinical symptomatology, showing that positive symptoms are a better explanation of individual differences in effective connectivity than negative symptoms in this patient sample (B) Patients with stronger positive symptoms (SAPS score) had more disinhibition within a DCM of pSTS.

We then tested for an association between psychopathology and effective connectivity in patients during social stimuli compared to non-social stimuli. Using parametric empirical Bayes (PEB), we compared the model evidence of a PEB-DCM with connection strengths explained by positive symptoms to the model evidence of a PEB-DCM explained by negative symptoms. Bayesian model comparison showed that between-patient differences in effective connectivity were better explained by their positive symptoms than by their negative symptoms. Inspection of the PEB model revealed that patients who reported a higher degree of positive symptoms had reduced intrinsic (inhibitory) coupling within a dynamic causal model of the posterior superior temporal sulcus (Posterior probability > 0.99). In other words, patients with more positive symptoms had more disinhibition within posterior superior temporal sulcus (pSTS) (Fig. 5).

## 4 Discussion

### 4.1 High-functioning FES patients have aberrant brain connectivity

In this study, we used DCM for fMRI to test for differences in effective connectivity between patients with first-episode schizophrenia and healthy matched controls during the HCP social cognition paradigm (Barch et al., 2013). This allowed us to identify pathophysiological differences in the feedforward and intrinsic connectivity in patients compared to controls. The behavioral results summarized in Table 2 suggest that these first-episode patients were high-functioning in relation to previous studies, where patients with schizophrenia typically performed 1–2 standard deviations below healthy controls on cognitive and social cognition tasks (Bora et al., 2009, Fatouros-Bergman et al., 2014, Fioravanti et al., 2012, Kern et al., 2004, Penn et al., 2008, Savla et al., 2013). This might be due to a short duration of illness, combined with a successful match of FES patients and controls. Despite their high level of functioning, our DCM results show aberrant brain connectivity in relation to healthy matched controls. Moreover, our results point to an association between cortical inhibition within the posterior superior temporal sulcus (pSTS) and the severity of positive symptoms in first-episode patients. Recent studies have shown differences in pSTS activation and functional connectivity with prefrontal cortex between patients with schizophrenia and healthy controls during different social perception paradigms (Backasch et al., 2013, Ciaramidaro et al., 2015, Jimenez et al., 2018, Mier et al., 2017, Okruszek et al., 2018). In line with our finding, Backash et al. showed an association between pSTS activation and delusional (positive) symptoms (Backasch et al., 2013). However, in contrast to these previous studies, we show an association between psychopathology and brain function using a biophysical model of the underlying neuronal activity, as opposed to the level of observed BOLD responses.

### 4.2 Active inference and failures of social inference

Computational theories of the brain that describe neuronal connectivity as a process of Bayesian inference are becoming increasingly useful for a mechanistic understanding of perception and action (Friston, 2010). In the context of social cognition, active inference offers a natural way to understand theory of mind as inferring the hidden states of another agent’s intentions, given their observed behavior (Friston and Frith, 2015). Crucially, this inference rests on a generative model of the mental states that cause a particular social behavior. For an agent to successfully engage in social communication, this generative model must be able to account for both the social behavior of other agents and one’s own behavior. In other word, one must model the behavior caused by other agents and the behavior caused by oneself and as being generated by the same model of intentions (Friston and Frith, 2015). We here provide an account of abnormal social cognition in terms of aberrant encoding of precision within a generative model of intentions or mental states.

In active inference, perception corresponds to inferring the hidden states in the world that cause sensory observations. These states are hidden in the sense that the world can only be observed through noisy sensory inputs. In order to infer the (hidden) mental states of other agents that cause a particular social behavior, the brain must have a generative model that combines prior beliefs about plausible mental states with the likelihood of observing a particular behavior to form posterior beliefs (theory of mind). These probabilistic beliefs are encoded in terms of their expectation and precision. Precision is simply the inverse variance or uncertainty with which the brain represents the external world. It follows from the form of this generative model that the brain must minimize the surprise $-\text{ln}\;p\left(\tilde{o}|m\right)$ about sensory observations $\tilde{o}$ at any one time, given a particular model *m* or explanation of those sensations. However, as computing surprise itself is mathematically intractable, a plausible solution is that the brain minimizes an upper bound on surprise known as variational free energy (Friston, 2010).

Feedback connections are thought to encode an agent’s internal predictions about hidden states in the external world, such as one’s beliefs about the mental states of other agents that constitute theory of mind (Friston and Frith, 2015). By contrast, feedforward connections mediate the ensuing prediction errors that are inconsistent with these predictions, given current sensory observations. The key imperative of active inference is to reduce uncertainty within the brain’s generative model of the world by actively sampling parts of the sensorium that require sensory interrogation (Schwartenbeck et al., 2019). When exposed to social stimuli, the influence of prediction errors on posterior beliefs (social inference) is controlled by their relative precision or confidence. Our results show that patients were less accurate in detecting social scenarios than healthy controls. At the same time, patients had increased feedforward connectivity from V5 to pSTS during these social stimuli compared to controls. This increased level of feedforward connectivity may be compensatory in nature and reflect a state where prediction errors are weighted by an abnormally high level of precision during visual stimuli. This fits well with theories proposing that schizophrenia is associated with abnormally high levels of prediction error during perceptual inference (Kapur, 2003). Our interpretation is that there is a failure to integrate the social information carried by prediction errors into a patient’s generative model of mental states that constitutes their theory of mind. In other words, there is an impairment in the way sensory information used to resolve uncertainty about the world during active sampling. Psychologically, this failure to resolve uncertainty about the world would result in a misinterpretation of social cues that may be understood as hypo-mentalizing (Bliksted et al., 2019). This interpretation is entirely supported by our finding that patients were less accurate in detecting social scenarios than healthy controls.

Neurobiologically, a developmental dysfunction of synaptic efficacy has been proposed as a likely disease mechanism in schizophrenia (McCutcheon et al., 2019). Specifically, a dysfunction of the glutamatergic *N*-methyl-*D*-aspartate (NMDA) receptor expressed at both excitatory pyramidal cells and GABAergic inhibitory interneurons (Murphy and Miller, 2003) has been proposed to play a central role in the generation of perceptual, cognitive and psychotic symptoms (Javitt, 2015, Javitt, 2010, Krystal et al., 1994, Krystal et al., 2017). Evidence from non-invasive electrophysiology in humans has been reported by Schmidt et al. who observed an increase in feedforward connectivity within the auditory system (Schmidt et al., 2013) and Rosch et al. who observed a selective disinhibition within the superior temporal gyrus, both under NMDA-receptor blockade with ketamine (Rosch et al., 2018). Similar findings under pharmacological manipulation of NMDA-receptor function have been linked to psychosis (Adams et al., 2013, Friston et al., 2016, Friston et al., 2014). Finally, Backash et al. who showed an association between pSTS activation and delusional (positive) symptoms (Backasch et al., 2013). Our finding of more disinhibition within pSTS in patients with positive symptoms concurs with these studies and adds to the evidence that psychosis may associated with an abnormal balance of excitation and inhibition (Jardri and Denève, 2013, O'Donnell, 2011).

### 4.3 Replicability and future research

Our reasons for using the HCP social cognition paradigm are twofold. First, it allowed us to replicate previous findings in the typical brain (Hillebrandt et al., 2014) using the exact same paradigm. Replicability in the normal population is valuable because it adds to the construct validity of our findings. Second, it allowed us to test for aberrant pathophysiology in a patient cohort using a standardized paradigm. Sharing of standardized stimulus paradigms and data analysis pipelines is essential for the replicability of neuroimaging findings in new datasets from both healthy and clinical cohorts across independent research sites. We are currently planning more research integrating fMRI with MEG and EEG in order to identify both differences and commonalities in pathophysiology across different subgroups of patients with schizophrenia, ranging from children at genetic risk of developing schizophrenia to first-episode patients.

## 5 Funding statement

This work was supported by “Seed Money” from the Interacting Minds Center, Aarhus University. MJD was funded by VELUX FONDEN [00013930].

## CRediT authorship contribution statement

**Martin J. Dietz:** Conceptualization, Investigation, Methodology, Formal analysis, Visualization, Writing - original draft, Writing - review & editing. **Yuan Zhou:** Conceptualization. **Lotte Veddum:** Investigation, Formal analysis. **Christopher D. Frith:** Conceptualization, Supervision. **Vibeke F. Bliksted:** Conceptualization, Investigation, Resources, Formal analysis, Project administration, Writing - original draft, Writing - review & editing.

## Declaration of Competing Interest

The authors declare that they have no known competing financial interests or personal relationships that could have appeared to influence the work reported in this paper.
